# Microwave-Assisted Synthesis of Visible Light-Driven BiVO_4_ Nanoparticles: Effects of Eu^3+^ Ions on the Luminescent, Structural, and Photocatalytic Properties

**DOI:** 10.3390/molecules30244757

**Published:** 2025-12-12

**Authors:** Dragana Marinković, Bojana Vasiljević, Nataša Tot, Tanja Barudžija, Sudha Maria Lis Scaria, Stefano Varas, Rossana Dell’Anna, Alessandro Chiasera, Bernhard Fickl, Bernhard C. Bayer, Giancarlo C. Righini, Maurizio Ferrari

**Affiliations:** 1 Vinča Institute of Nuclear Sciences, National Institute of the Republic of Serbia, University of Belgrade, P.O. Box 522, 11001 Belgrade, Serbia; bojana.vasiljevic@vin.bg.ac.rs (B.V.); natasa.tot@vin.bg.ac.rs (N.T.); tbarudzija@vin.bg.ac.rs (T.B.); 2 Institute of Photonics and Nanotechnologies (IFN CNR, CSMFO Laboratory) and FBK Photonics Unit, Via alla Cascata 56/C, Povo, 38123 Trento, Italy; sscaria@fbk.eu (S.M.L.S.); stefano.varas@cnr.it (S.V.); dellanna@fbk.eu (R.D.); chiasera@fbk.eu (A.C.); 3 Fondazione Bruno Kessler, Center for Sensors and Devices (FBK-SD), Via Sommarive, 18, Povo, 38123 Trento, Italy; 4 Institute of Materials Chemistry, Technische Universität Wien (TU Wien), Getreidemarkt 9/165, A-1060 Vienna, Austria; bernhard.fickl@tuwien.ac.at (B.F.); bernhard.bayer-skoff@tuwien.ac.at (B.C.B.); 5 Nello Carrara Institute of Applied Physics (IFAC CNR), Sesto Fiorentino, 50019 Firenze, Italy; righini@ifac.cnr.it

**Keywords:** microwave-assisted synthesis, BiVO_4_, Eu^3+^-doping, tetragonal structure, monoclinic structure, degradation of Rhodamine B, luminescence, micro-Raman, decay dynamics, photocatalytic properties

## Abstract

The optimization of BiVO_4_-based structures significantly contributes to the development of a global system towards clean, renewable, and sustainable energies. Enhanced photocatalytic performance has been reported for numerous doped BiVO_4_ materials. Bi^3+^-based compounds can be easily doped with rare earth (RE^3+^) ions due to their equal valence and similar ionic radius. This means that RE^3+^ ions could be regarded as active co-catalysts and dopants to enhance the photocatalytic activity of BiVO_4_. In this study, a simple microwave-assisted approach was used for preparing nanostructured Bi_1−x_Eu_x_VO_4_ (x = 0, 0.03, 0.06, 0.09, and 0.12) samples. Microwave heating at 170 °C yields a bright yellow powder after 10 min of radiation. The materials are characterized through X-ray diffraction (XRD), transmission electron microscopy (TEM), ultraviolet–visible–near-infrared diffuse reflectance spectroscopy (UV-Vis-NIR DRS), photoluminescence spectroscopy (PL), and micro-Raman techniques. The effects of the different Eu^3+^ ion concentrations incorporated into the BiVO_4_ matrix on the formation of the monoclinic scheelite (*ms*-) or tetragonal zircon-type (*tz*-) BiVO_4_ structure, on the photoluminescent intensity, on the decay dynamics of europium emission, and on photocatalytic efficiency in the degradation of Rhodamine B (RhB) were studied in detail. Additionally, microwave chemistry proved to be beneficial in the synthesis of the *tz*-BiVO_4_ nanostructure and Eu^3+^ ion doping, leading to an enhanced luminescent and photocatalytic performance.

## 1. Introduction

Recent advances in modern technology indicate an intense acceleration in the nanomaterials research area. In their ongoing effort to expand the boundaries of what is now possible, scientists are increasingly focusing on developing valuable semiconducting nanomaterials [1,2]. Their involvement in photocatalysis applications, such as self-cleaning coatings, H_2_ production, CO_2_ conversion, antibacterial treatments, and water remediation, has garnered significant attention over the past decade. However, a number of crucial factors, including light absorption efficiency, charge carrier recombination, photocatalyst stability, and scalability, need to be optimized to improve photocatalytic activity. To overcome this restriction, researchers have looked into doping photocatalysts, especially TiO_2_, with metals or non-metals [3] and creating hybrid materials with ferrites, Metal–Organic Frameworks (MOFs), and perovskites, which show enhanced light absorption throughout the visible spectrum [4,5,6]. In the case of rapid electron–hole recombination, methods to improve photocatalytic efficiency include coupling semiconductor materials to form heterojunctions, incorporating co-catalysts, and designing Z-scheme photocatalysts [7,8]. Additionally, certain photocatalytic materials, particularly some metal oxides, are inappropriate for use due to their potential toxicity and environmental persistence [9,10]. However, the negative environmental impact could be reduced by employing eco-friendly techniques, such as the creation of bio-based photocatalysts or the utilization of non-toxic materials [11,12]. Nevertheless, improvements in economical synthesis techniques are crucial for the large-scale manufacturing of photocatalysts.

One of the finest bismuth-based semiconducting materials is bismuth vanadate (BiVO_4_) due to its non-toxic nature, distinct physical and chemical properties, and good response to visible light excitation [13,14,15,16,17]. Additionally, various morphologies of BiVO_4_ have been developed, exhibiting an excellent visible light photocatalytic efficiency in degrading wastewater contaminants [4,5,6,7]. However, the limited separation (higher recombination rate) and transport capabilities of photogenerated electron–hole pairs severely restrict the commercial-scale application of BiVO_4_. Overcoming these limitations through surface modification, morphology control, or doping elements can enhance the photocatalytic performance of this valuable nanomaterial [18,19,20].

Rare earth ions are crucial in several applications in nanotechnology [21] and photonics, for instance, as a luminescent thermometer [22,23,24] or as a bioimaging contrast agent [25,26]. Doping rare earth metals into Bi-based photocatalysts has been demonstrated to enhance their photocatalytic activity effectively [20]. In fact, the lanthanide elements, with their partially filled 4f electronic orbits, can participate in the electronic structure, effectively capturing photogenerated electrons (e^−^) or holes (h^+^), further blocking carrier recombination. Additionally, rare earth ions possess remarkable upconversion properties, enabling the transformation of near-infrared light into visible light [27]. In the case of BiVO_4_, several studies have reported substituting Bi^3+^ with Nd [23,28] or Gd [29,30], or co-doping with trivalent lanthanide ions such as Yb/Tm [31], Nd/Er [32], Yb/Er [22], Er/Tm/Yb [33,34], or Tm/Er, Yb, Y [35]. Rare earth doping, such as Eu^3+^ ions in BiVO_4_, has emerged as a promising strategy to enhance the photocatalytic performance of this material [36]. Using the hydrothermal approach, Zhang et al. produced Eu-doped BiVO_4_ with a maximum Eu-doping concentration of 7.30 wt%, resulting in the maximum degradation of methyl orange (MO) [37]. Additionally, Shan et al. confirmed that Eu-doped BiVO_4_ showed a higher photocatalytic performance in degrading RhB and methylene blue (MB) compared to bare BiVO_4_ [38]. On the other hand, BiVO_4_ co-doped with Eu/B [39] and KCl [40] exhibited synergistic effects, attributed to an increased surface area and enhanced separation efficiency of photogenerated charge carriers. Some tri-modified BiVO_4_ photocatalysts (co-doped with Ag, B, and Eu) have also shown an enhanced catalytic performance in degrading methyl orange (MO) and tetracycline (TC) [41]. Worthy of note, such improved semiconducting materials exhibit a noticeable phase modification, as demonstrated by Dhakal et al. In the case of Yb^3+^/Tm^3+^ co-doped BiVO_4_, studies have shown that doping promotes a high proportion of the *tz*-phase, approximately 80%, resulting in a marked enhancement of photocatalytic efficiency compared to undoped *ms*-BiVO_4_ [42]. The electronic structure of the Eu^3+^ ions is defined by its 4*f*–4*f* electron transitions, which result in sharp, characteristic peaks with red luminescence. The Eu^3+^ ions’ local environment and its interactions with the BiVO_4_ material significantly influence its photocatalytic performance. The Eu^3+^ ions can act as an electron trap to separate photogenerated electrons and holes in a BiVO_4_ material due to enhanced charge carrier dynamics. The different pollutants’ interactions with Eu^3+^ ions in BiVO_4_ via f-orbitals increase its ability to absorb the pollutant molecules and enhance photocatalytic efficiency [15].

The hydrothermal method is widely employed for synthesizing rare earth-doped Bi-based photocatalysts, and the microwave-assisted synthesis of these nanomaterials has been reported only in a limited number of studies [34,42,43]. There are several advantages in the use of microwave-assisted synthesis. Microwave irradiation plays a crucial role by accelerating reaction kinetics, enabling rapid initial heating and ultimately increasing reaction rates. This approach yields cleaner products, promotes the faster consumption of starting materials, and enhances the overall yield [44,45]. Furthermore, uniform heating and a better control over process parameters contribute to a greater reproducibility of reaction conditions and constitute another pro of this technique [46,47]. However, in the synthesis of nanomaterials, the microwave heating method is a novel and uniform heating synthesis technique whose advancement should be pursued, as the use of greener reaction media significantly reduces chemical waste and reaction times [48,49].

In this study, we employed a simple microwave-assisted approach to prepare nanostructured Bi_1−x_Eu_x_VO_4_ (x = 0, 0.03, 0.06, 0.09, and 0.12) samples. The combined effects of nanostructure and Eu^3+^ ion doping enhanced luminescent and photocatalytic performance. This approach not only optimizes the photocatalytic activity in the degradation of RhB and deepens the understanding of the role of Eu^3+^ doping in improving optical properties, but also induces the formation of both *ms*- and/or *tz*-type BiVO_4_ structures. Both obtained structures are of great importance in terms of their photocatalytic performances and applications for the degradation of organic contaminants [14,15].

## 2. Results

### 2.1. Structural and Morphological Properties of the Bi_1−x_Eu_x_VO_4_ (x = 0, 0.03, 0.06, 0.09, and 0.12) Samples

#### 2.1.1. X-Ray Diffraction (XRD) Analysis

**Figure 1:**
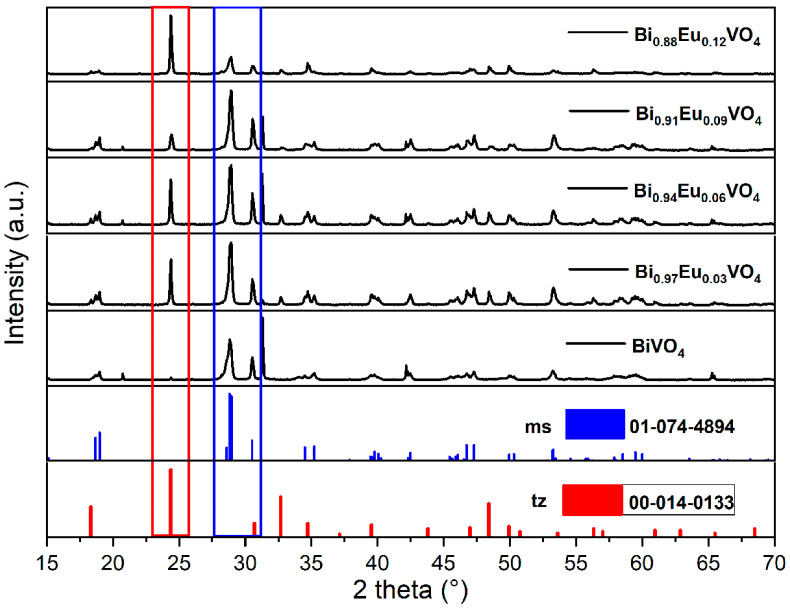
XRD patterns for Bi_1−x_Eu_x_VO_4_ (x = 0, 0.03, 0.06, 0.09, and 0.12) samples together with vertical bars from card references (No. 01-074-4894) for *ms*-BiVO_4_ and (No. 00-014-0133) for *tz*-BiVO_4_.

The powder samples were yellow; their crystalline structures were identified through XRD analysis. The XRD patterns, together with card references (JCPDS Card No. 01-074-4894) for *ms*-BiVO_4_ and (JCPDS Card No. 00-014-0133) for *tz*-BiVO_4_, are presented in Figure 1.

The XRD pattern of undoped BiVO_4_ (Bi_1−x_Eu_x_VO_4_ (x = 0)) synthesized via microwave-assisted synthesis matches well with that of the standard bulk monoclinic clinobisvanite crystalline system (space group: I_2/b_; a = 5.1935, b = 5.0898, c = 11.6972 Å, and β = 90.3871°). The peaks at approximately 2θ = 18.9°, 29.1°, 30.5°, 35.2°, and 39.7° are assigned to the (011), (112), (004), (020), and (211) crystal planes, respectively, confirming the high crystallinity of the microwave-prepared *ms*-BiVO_4_ powder.

In the case of Eu^3+^-doped BiVO_4_ samples, Bi_1−x_Eu_x_VO_4_ (x = 0.03, 0.06, 0.09, and 0.12), the XRD patterns show the presence of a mixture of *ms*-BiVO_4_, (JCPDS Card No. 01-074-4894), and *tz*-BiVO_4_ (JCPDS Card No. 14–00133) phases. The main peaks at approximately 2θ = 29.1° and 2θ = 24.3° are characteristic peaks of the *ms*- and *tz*-phases, respectively. In addition, the relative intensity of the *ms*-related peak at approximately 29.1° decreases with an increasing content of the Eu^3+^ ions. In contrast, the relative intensity of the *tz*-related peak at approximately 24.3° increases with increases in the content of the Eu^3+^ ions, so that the *tz*-type structure becomes dominant in the sample Bi_1−x_Eu_x_VO_4_ (*x* = 0.12). This phase transition may be attributed to the stability of the lattice constant of BiVO_4_ when Bi^3+^ ions (ionic radius = 1.17 Å, coordination VIII) are substituted with Eu^3+^ ions (ionic radius = 1.066 Å, coordination VIII). The literature overview of the formation of the *ms*- or *tz*-crystalline phase in the Bi_1−x_Eu_x_VO_4_ samples in comparison with the results obtained in this work is given later in the Section on “The Formation of *ms*- or *tz*-Crystalline Phase in Bi_1−x_Eu_x_VO_4_ (x = 0, 0.03, 0.06, 0.09, and 0.12) Samples”.

The crystallite size was determined using Scherrer’s equation (Equation (1)), applied to the most intense peak in the XRD pattern [50]:

(1) $$D=\frac{\left.k\lambda \right.}{\beta cos\theta }$$

where D is the crystallite size, k represents the shape factor constant with value of 0.9, λ is the wavelength of the X-ray radiation (0.15406 nm), β is the full-width half-maximum (FWHM), and θ is Bragg’s angle of reflection. With reference to the peak of maximum intensity at the 2θ diffraction angle of 29.1°, the average crystallite size for microwave-synthesized *ms*-BiVO_4_ was found to be 16 nm according to this equation. The crystallite size increases with the Eu^3+^ ion content, reaching up to 55 nm for Bi_0.88_Eu_0.12_VO_4_, as determined at a 2θ diffraction angle of 24.3°, corresponding to the dominant *tz*-BiVO_4_ phase. The main aim was to obtain an optimized *tz*-BiVO_4_ nanostructure, which was recently presented in published papers as a structure exhibiting better photocatalytic performance than *ms*-BiVO_4_ with respect to pollutant removal [14,15].

#### 2.1.2. Transmission Electron Microscopy (TEM)

**Figure 2:**
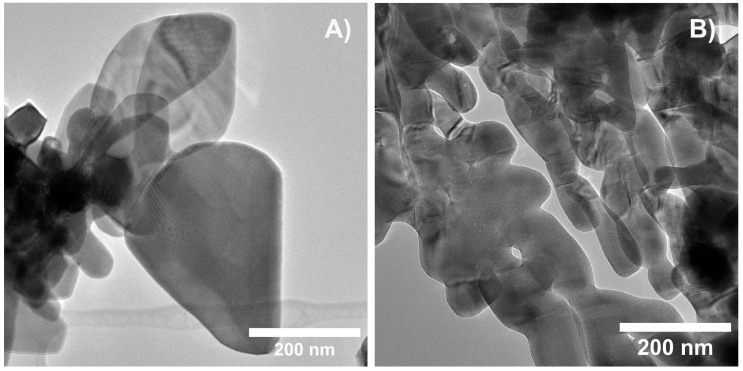
Representative TEM images for (**A**) BiVO_4_ and (**B**) Bi_0.88_Eu_0.12_VO_4_ samples.

Representative TEM images for BiVO_4_ and Bi_0.88_Eu_0.12_VO_4_ samples are given in Figure 2A,B, respectively. Both samples consist of well-crystallized irregular spheroidal nanoparticles with a size of approximately 20–50 nm. The particle size assessed using TEM is compatible with the crystallite size obtained from XRD measurements, suggesting that each particle comprises a single crystallite. Additionally, the majority of the synthesized nanoparticles show some degree of aggregation, creating large nanoparticles with an average diameter of about 100 nm, which is frequently seen for BiVO_4_ nanoparticles because of their high surface energy [16,51]. For microwave-synthesized processes, it was found that the morphology and size of nanostructures were strongly dependent on the heating method and temperature. Under intensive microwave heating, nanoparticles subsequently self-assembled to form aggregated nanostructures from the aqueous solution [52].

### 2.2. Optical Properties of the Bi_1−x_Eu_x_VO_4_ (x = 0, 0.03, 0.06, 0.09, and 0.12) Samples

#### 2.2.1. Fourier Transform Infrared Spectroscopy (FTIR)

**Figure 3:**
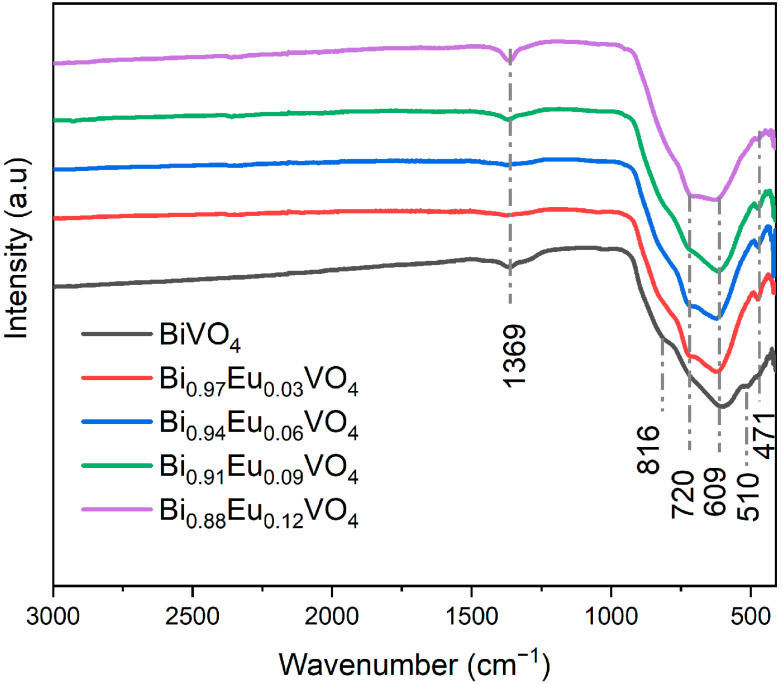
FTIR spectra for the Bi_1−x_Eu_x_VO_4_ (x = 0, 0.03, 0.06, 0.09, and 0.12) samples.

To identify the functional groups in the prepared Bi_1−x_Eu_x_VO_4_ (*x* = 0, 0.03, 0.06, 0.09, and 0.12) samples, the FTIR spectra were measured, and are presented in Figure 3.

The FTIR analysis of undoped BiVO_4_ shows the characteristic peaks of BiVO_4_ in the range of around 500–820 cm^−1^. A strong absorption band at 609 cm^−1^ with a shoulder at 816 cm^−1^ is associated with the asymmetric and symmetric stretching vibrations of the VO_4_^3−^ tetrahedron, respectively. Additionally, the peak observed at 510 cm^−1^ is assigned to the Bi–O bond stretching vibrations.

On the other side, the FTIR analysis of Bi_1−x_Eu_x_VO_4_ (x = 0.03, 0.06, 0.09, and 0.12) samples shows the same characteristic peaks as undoped BiVO_4_ in the range 500–820 cm^−1^, while two new peaks appear at 471 cm^−1^ and 720 cm^−1^ assigned to Eu-O stretching vibrations. A small peak observed in all samples at 1369 cm^−1^ can be assigned to nitrates from the nitric acid used during the synthesis or from Bi(NO_3_)_3_. These results are also in agreement with the previously reported literature [53].

#### 2.2.2. Diffuse Reflectance Spectroscopy (DRS)

**Figure 4:**
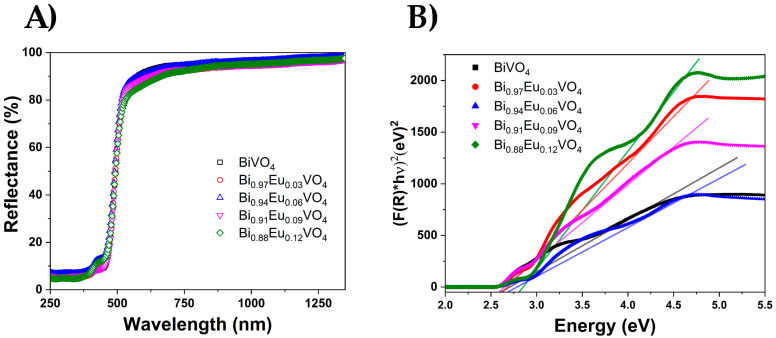
(**A**) The diffuse reflectance spectra and (**B**) energy dependence of (FKM(R)hν)^2^ for Bi_1−x_Eu_x_VO_4_ (x = 0, 0.03, 0.06, 0.09, and 0.12) samples.

The diffuse reflectance spectra of Bi_1−x_Eu_x_VO_4_ samples presented in Figure 4A are used to estimate energy band gaps through Tauc’s plot method (Figure 4B). The absorption observed below 350 nm for all studied samples is assigned to the absorption of vanadate groups. An additional weak peak observed at approximately 424 nm in the Bi_1−x_Eu_x_VO_4_ samples is attributed to the electronic transitions of Eu^3+^ ions. Figure 4B presents the energy dependence of (FKM(R)hν)^2^ for Bi_1−x_Eu_x_VO_4_ samples.

The band gap, Eg, was estimated from the absorption edge wavelength of the inter-band transition according to the following equation:

(2) $${\left(FKM\left(R\right)\;\times \;h\nu \right)}^{n}=A\;(h\nu -Eg)$$

where FKM(R) is the Kubelka–Munk function, with FKM(R) = (1 − R)^2^/2R; R is the observed reflectance in the UV–vis spectra; *n* = 2 for a direct allowed transition and n = 1/2 for an indirect allowed transition; A is a proportionality constant; and hν is the photon energy. BiVO_4_ is a direct gap semiconductor; therefore, *n* = 2 [54,55]. According to Equation (2), the Eg values were determined by extrapolating the linear portion of the (FKM(R)hν)^2^ curve to the intersection with the *X*-axis. As can be seen in Figure 4B, the optical band gap shifts to a higher energy with increasing Eu^3+^ contents. The Eg values, ranging from approximately 2.55 to 2.80 eV, are in full agreement with recently published results [56]. 

#### 2.2.3. Optical Absorption

**Figure 5:**
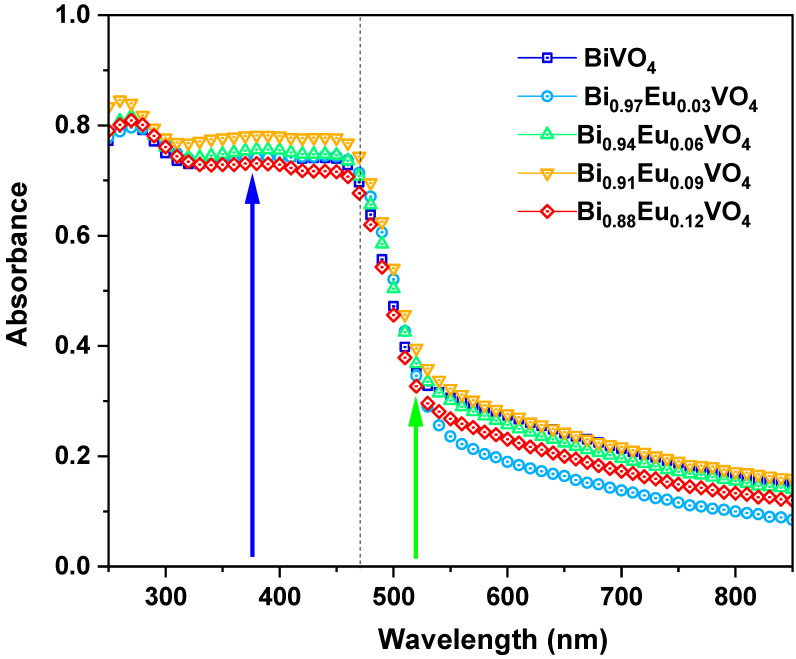
Absorption spectra of Bi_1−x_ Eu_x_VO_4_ (*x* = 0, 0.03, 0.06, 0.09, and 0.12) powder samples. Arrows indicate 375 nm (blue) and 518 nm (green).

The UV–visible absorption spectra of Bi_1−x_Eu_x_VO_4_ (x = 0, 0.03, 0.06, 0.09, and 0.12) powder samples are shown in Figure 5. The arrows indicate 375 nm (blue) and 518 nm (green). The samples exhibit an absorption band in the UV and visible light range, with the absorption edge at 470 nm. The absorption is stronger in the ultraviolet (UV) region, with an absorption tail extending up to 800 nm. The absorption curves display a sharp decrease in absorbance at the transition region between UV and visible wavelengths, as highlighted by the dashed line in Figure 5. The Eu^3+^-doped samples exhibit a slight reduction in absorption within the visible region (>500 nm) compared to the undoped BiVO_4_.

#### 2.2.4. Photoluminescence (PL) Emission Measurements Using Continuous-Wave Lasers

**Figure 6:**
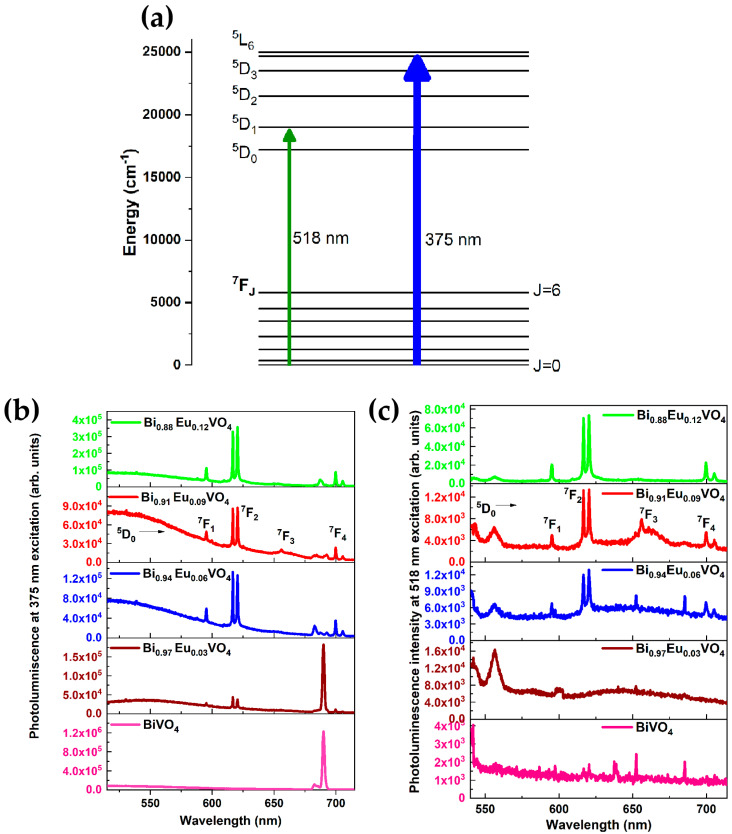
(**a**) Energy level diagram of Eu^3+^. The blue and green arrows indicate the excitation wavelengths at 375 nm and 518 nm, respectively. PL emission spectra of Bi_1−x_Eu_x_VO_4_ (x = 0, 0.03, 0.06, 0.09, 0.12) samples at (**b**) 375 nm and (**c**) 518 nm excitation wavelength. For the 0.09 mol.% of Eu^3+^ in the BiVO_4_ sample, ^5^D_0_ → ^7^F_J_ (J = 1–4) transitions are labeled for easy identification.

Figure 6a shows the energy level of the Eu^3+^ ion. The two excitations at 375 nm and 518 nm are indicated by the blue and green arrows, respectively. The photoluminescence (PL) emission spectra of the undoped and Eu^3+^-doped BiVO_4_ samples for the excitation wavelengths of 375 nm and 518 nm are presented in Figure 6b,c, respectively. For Eu^3+^-doped BiVO_4_ samples, peaks corresponding to typical europium emission, i.e., ^5^D_0_ → ^7^F_J (J = 1–4)_ transitions, are present, with the ^5^D_0_ → ^7^F_2_ transition being the dominant one for both 375 nm and 518 nm excitation wavelengths. The emission spectrum shows a gradual increase in the intensity value with the increase in Eu^3+^ doping. These sharp spectral features of the typical Eu^3+^ emission plot indicate that the europium ions are uniformly doped in the BiVO_4_ matrix [57]. For Eu^3+^-doped BiVO_4_ samples under both 375 nm and 518 nm excitation, a peak is present at ~595.5 nm: this indicates the ^5^D_0_ → ^7^F_1_ transition corresponding to magnetic dipole (MD) transition. Intense and clearly resolved peaks at 616 nm indicate a ^5^D_0_ → ^7^F_2_ transition corresponding to an electric dipole (ED) transition. In addition to this, ^5^D_0_ → ^7^F_3,4_ transition peaks are also present. When the samples are excited, in addition to the charge transfers of orbitals O 2p, V 3d, and Bi 6s, the transfer of energy to Eu^3+^ ions results in the PL emission [58]. The narrow peak at 690 nm for 375 nm excitation is a spurious structure; similarly, the broad emission after 550 nm is due to the background emission from the cuvette. The f-f transitions in europium involve electron jumps within the shielded 4f subshell of the Eu^3+^ ion, leading to sharp, characteristic UV-Vis absorption and emission spectra. While f-f transitions are formally “forbidden” by spin and parity selection rules, their intensities can be significantly enhanced by the ligand environment, especially through asymmetry in the coordination sphere [36,59,60].

#### 2.2.5. Time-Resolved Fluorescence Spectroscopy (TRS)

**Figure 7:**
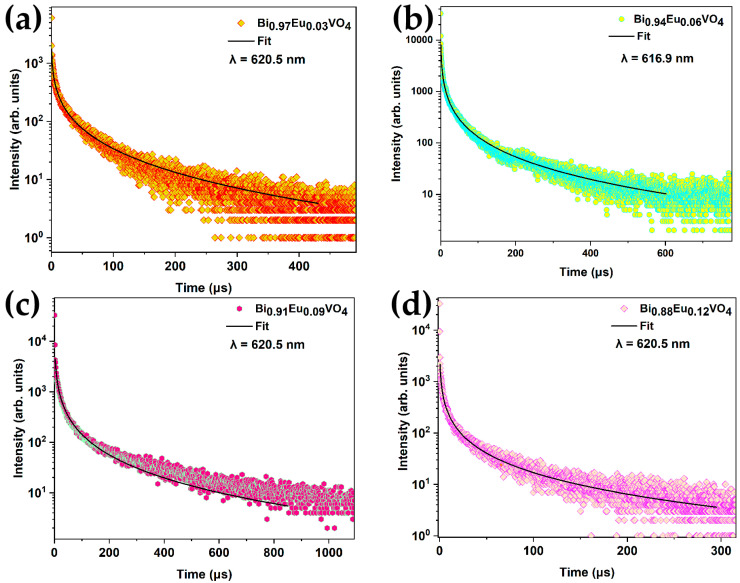
Measured and fitted the time-resolved PL decay curves corresponding to ^5^D_0_ ⟶ ^7^F_2_ transition (620.5 nm) for (**a**) Bi_0.97_Eu_0.03_VO_4_, (**b**) Bi_0.94_Eu_0.06_VO_4_, (**c**) Bi_0.91_Eu_0.09_VO_4_, and (**d**) Bi_0.88_Eu_0.12_VO_4_ samples. Pulsed excitation was at 355 nm.

Time-resolved photoluminescence measurements were performed to study the decay dynamics of europium emission in the BiVO_4_ matrix. For this purpose, the sample was excited using the second harmonic (355 nm) of a Nd:YAG pulsed laser (repetition rate ~10 Hz, pulse width ~8 ns), and the PL emission was collected using a home-built collection geometry configured with a time-resolved setup and multi-channel counters [58]. The PL spectra obtained for Bi_1−x_Eu_x_VO_4_ samples display dominant emissions corresponding to the ^5^D_0_ → ^7^F_2_ transition, whereas BiVO_4_ does not show any emissions. The measured and fitted time-resolved PL decay curves corresponding to the ^5^D_0_ ⟶ ^7^F_2_ transition for all Eu^3+^ samples are shown in Figure 7. In general, the decay curves follow stretched exponential decay characteristics because of the local environment of Eu^3+^ in the BiVO_4_ matrix [61]. Here, relaxation refers to the active non-radiative channels, such as quenching centers in the lattice, possible energy migration among the activators, and surface localization. Hence, the stretched exponential or Kohlrausch model [57,61,62] is used to fit the decay curves as given by the following equation:

(3) $$\varphi \left(t\right)=\;\varphi \left(0\right){exp[-\left(\frac{t}{\tau }\right)}^{\beta }]$$

where 0<β < 1 is the stretching parameter and τ is the radiative lifetime.

The radiative lifetime τ for Eu^3+^-doped samples corresponding to the ^5^D_0_ ⟶ ^7^F_2_ transition is obtained from the fit of the decay curves in Figure 7a–d using Equation (3) and is reported in Table 1. The decay curves of Eu^3+^ exhibit a non-single exponential behavior. Evaluating the lifetime is particularly challenging when systems display such behavior, as it often arises from multiple, competing relaxation processes [63,64]. Table 1 presents both the 1/e lifetime, defined as the time after the excitation pulse at which the light intensity decreases to 1/e of its initial value, and the so-called average lifetime, given by the following equation:

$$\frac{\int_{0}^{\infty }t\;I\left(t\right)dt}{\int_{0}^{\infty }I\left(t\right)dt}$$

The 1/e lifetime is especially useful in photonic engineering applications, as it enables a direct comparison among different hosts and systems. It should be noted, however, that the average lifetime is strongly influenced by the long-time decay behavior and has limited significance in cases involving multiple competitive relaxation processes, such as in the present study [64].

The lifetime τ can be evaluated correctly only for the sample with the lowest Eu^3+^ content. As Eu^3+^ doping increases, the lifetime drops quickly with a very fast decay; similar behavior has been observed in BiVO_4_ nanoparticles activated by Eu^3+^ ions [65]. This deviation is attributed to the nonradiative energy transfer from the excited states to quenching centers within the lattice, as well as possible energy migration among activator ions. These energy losses introduce additional decay pathways, resulting in the observed nonexponential decay profiles that are correctly fitted by a stretched exponential. The contribution from surface-localized Eu^3+^ in BiVO_4_ should also be considered. In fact, the lifetime of surface-localized Eu^3+^ in BiVO_4_ is notably shorter than the typical millisecond-scale lifetime of Eu^3+^ ions in host lattices [65]. As above, the deviation is attributed to a nonradiative energy transfer to some quenching centers in the lattices or possible energy migrations among the activators [51]. The excitation energy losses provide some extra decay channels, inducing the nonexponential decay natures.

#### 2.2.6. Micro-Raman Spectroscopy Measurements

**Figure 8:**
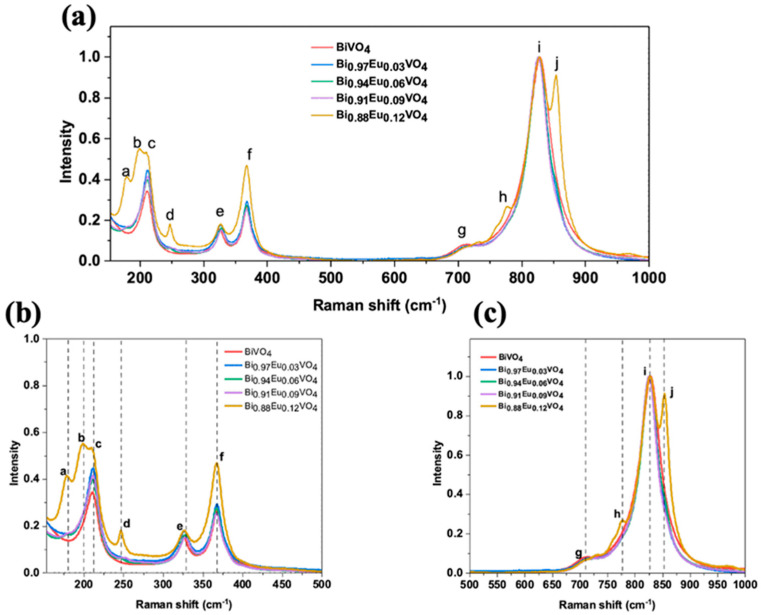
(**a**) Raman spectra of Bi_1−x_Eu_x_VO_4_ (x = 0.0, 0.03, 0.06, 0.09, and 0.12) samples. For more clarity, the zoomed-in views of the spectra are shown as (**b**,**c**).

The Raman spectra of undoped and Eu^3+^-doped BiVO_4_ exhibit characteristic peaks, labeled with letters as shown in Figure 8a and marked with vertical lines in the magnified section views of Figure 8b,c for clarity. The assigned letters correspond to the following wavenumbers: (a) 179 cm^−1^; (b) 197 cm^−1^; (c) 211 cm^−1^; (d) 247 cm^−1^; (e) 327 cm^−1^; (f) 367 cm^−1^; (g) 708 cm^−1^; (h) 778 cm^−1^; (i) 829 cm^−1^; and (j) 854 cm^−1^. The XRD analysis confirms that the samples comprise a mixture of *ms*-BiVO_4_ and *tz*-BiVO_4_ phases. Consequently, Raman modes corresponding to both phases are present, as summarized in Table 2.

Figure 8 further highlights Raman peaks that track the structural phase evolution with increasing Eu doping. The peak at ~211 cm^−1^ (c in the Figures), present across all samples, including the undoped one, corresponds to an external lattice mode common to both phases. In contrast, the two peaks at 179 cm^−1^ (a) and 197 cm^−1^ (b), which appear predominantly at higher Eu concentrations (especially at x = 0.12), are characteristic of monoclinic distortions or phase coexistence. The increasing intensity of these lower-frequency peaks with Eu content indicates enhanced lattice distortion and possibly the persistence of local monoclinic-like environments, despite overall doping-induced structural changes. The 247 cm^−1^ peak (d), primarily associated with Bi–O stretching vibrations in the tetragonal phase, is absent in the undoped sample but becomes prominent at higher doping, confirming the phase transition. The peak shifts near 254 cm^−1^ at x = 0.06 suggest lattice distortion linked to coexistence with a dominant monoclinic phase and strain.

Monoclinic phase signatures include peaks at 708 cm^−1^ (g) and 829 cm^−1^ (i) corresponding to antisymmetric and symmetric V–O stretching modes, respectively. These peaks decrease in intensity at x = 0.12, while tetragonal-phase peaks at 778 cm^−1^ (h) and 854 cm^−1^ (j) increase, marking the phase transition. Intermediate doping levels (x = 0.06 and 0.09) exhibit a coexistence of both phases, as evidenced by the simultaneous presence of monoclinic and tetragonal peaks with varying intensities.

Several peaks display doping-dependent shifts. The 327 cm^−1^ peak (e), related to the asymmetric deformation of the VO_4_^3−^ tetrahedron, shifts to higher wavenumbers up to x = 0.09, likely due to the substitution of Bi by smaller Eu ions, causing lattice contraction. This shift diminishes at x = 0.12, possibly due to lattice softening as the monoclinic phase diminishes. The 367 cm^−1^ (f) and 829 cm^−1^ (j) peaks also show a variable red shift indicative of doping-induced strain or disorder [59,62,66,67]. The shifts observed at (b) and (c) in the Raman spectra (together with the XRD results) confirm that the dopants caused distortions in the BiVO_4_ crystal lattice. These distortions likely happened by replacing Bi^3+^ with Eu^3+^ ions [59]. On the other hand, if Eu^3+^ were replacing V^5+^ instead, a shift toward lower wavenumbers should be expected, as Eu^3+^ has a greater atomic mass than V^5+^, according to [66,68].

Overall, the Raman spectral evolution clearly demonstrates a doping-driven structural transition from monoclinic to tetragonal BiVO_4_, with associated shifts reflecting changes in lattice dynamics and phase (Table 2).

### 2.3. Photocatalytic Activity of Bi_1−x_Eu_x_VO_4_ (x = 0, 0.03, 0.06, 0.09, 0.12) Samples

**Figure 9:**
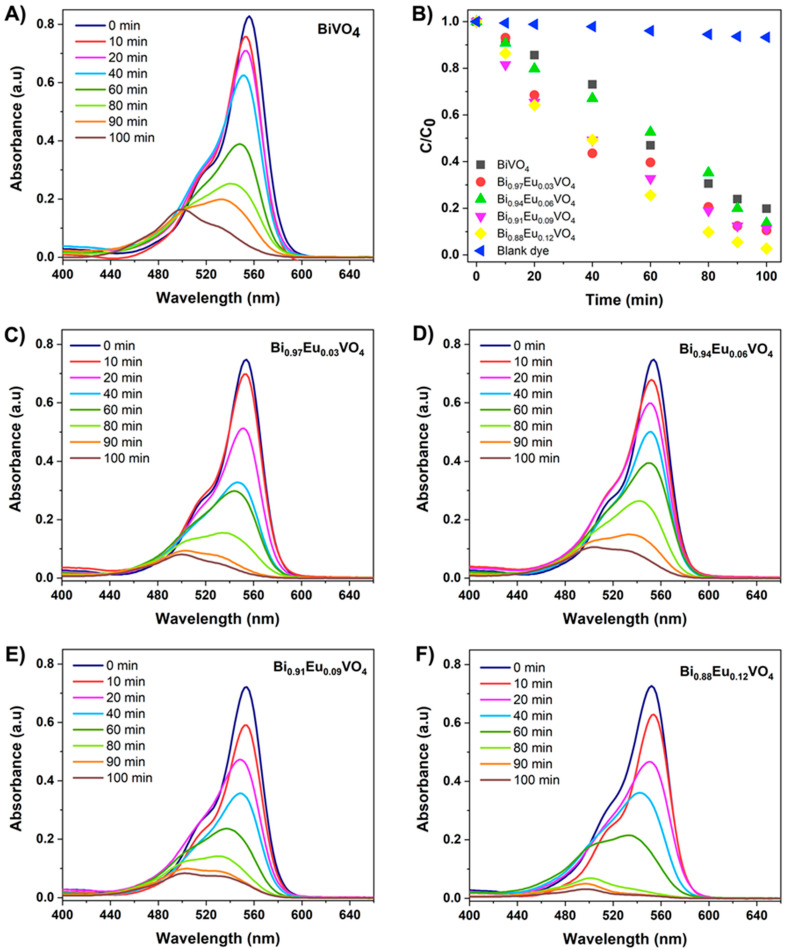
UV-Vis absorption spectra over irradiation time for photocatalytic degradation of 5 ppm RhB in the presence of 40 mg of (**A**) BiVO_4_, (**C**) Bi_0.97_Eu_0.03_VO_4_, (**D**) Bi_0.94_Eu_0.06_VO_4_, (**E**) Bi_0.91_Eu_0.09_VO_4_, and (**F**) Bi_0.88_Eu_0.12_VO_4_ photocatalyst and (**B**) the corresponding photocatalytic degradation rate curves for blank dye (RhB) and all microwave-synthesized Bi_1−x_Eu_x_VO_4_ (x = 0, 0.03, 0.06, 0.09, 0.12) photocatalysts.

The photodegradation of RhB was employed to evaluate the photocatalytic activities of all microwave-synthesized Bi_1−x_Eu_x_VO_4_ samples. UV-Vis absorption spectra over irradiation time for the photocatalytic degradation of 5 ppm RhB in the presence of 40 mg of the appropriate Bi_1−x_Eu_x_VO_4_ photocatalyst are presented in Figure 9. The change in the absorption spectra of the RhB solution during the photodegradation process at different irradiation times is presented in Figure 9A for the undoped BiVO_4_ photocatalyst. The absorbance of RhB at 554 nm decreased gradually after 100 min of irradiation in the presence of undoped BiVO_4_, indicating that microwave-synthesized catalysts are effective for dye removal in wastewater treatment. Compared with the undoped BiVO_4_, the RhB photocatalytic degradation shows lower absorbance intensities as the concentration of the Eu^3+^-dopant increases (Figure 9C–F). The removal efficiency curves presented in Figure 9B illustrate the relative concentration of the RhB solution over irradiation time for blank dye (RhB) and all microwave-synthesized Bi_1−x_Eu_x_VO_4_ (x = 0, 0.03, 0.06, 0.09, and 0.12) photocatalysts. In contrast to the undoped BiVO_4_, with a removal efficiency of 81% within 100 min of irradiation, Eu^3+^-doped BiVO_4_ samples exhibited higher efficiencies of 89%, 87%, and 91% for Bi_0.97_Eu_0.03_VO_4_, Bi_0.94_Eu_0.06_VO_4_, and Bi_0.91_Eu_0.09_VO_4_, respectively. The degradation rate of 97% for RhB dye was observed in the highest Eu^3+^-doped BiVO_4_ sample, Bi_0.88_Eu_0.12_VO_4_.

It can be noted that, in comparison with the undoped BiVO_4_, the Eu^3+^-doped BiVO_4_ photocatalysts exhibit a much higher photocatalytic activity in the degradation of RhB under visible light irradiation. There are two different possibilities for the enhanced photocatalytic performances with increasing concentrations of Eu^3+^ ions in the BiVO_4_ matrix: (i) an appropriate amount of Eu^3+^ ions can improve the separation efficiency of photogenerated electron–hole pairs and hinder their recombination, and (ii) the dominant *tz*-BiVO_4_ structure exhibited a better photocatalytic performance than *ms*-BiVO_4_ with respect to RhB removal.

To gain a deeper insight into the photocatalytic behavior, the kinetics of RhB degradation were analyzed using the Langmuir–Hinshelwood pseudo-first-order model. The calculated kinetic parameters are summarized in Table 3.

Pristine BiVO_4_ shows a rate constant of 1.67 × 10^−2^ min^−1^, while Eu^3+^-doped samples exhibit enhanced performance, reaching 3.46 × 10^−2^ min^−1^ for Bi_0.88_Eu_0.12_VO_4_, which is more than twice the value obtained for undoped BiVO_4_. This increase in the kinetic constant follows the same trend as the removal efficiencies, confirming that Eu^3+^ ions accelerate the photocatalytic reaction. A similar relationship between structural modification and kinetic enhancement has been reported in the literature. Ren et al. demonstrated that a 3 wt% N-CQDs/UBWO composite achieves a kinetic constant of 0.0409 min^−1^, almost four times higher than pristine Bi_2_WO_6_ (0.01628 min^−1^) under visible light irradiation [69]. In RhB degradation systems, Ag-modified BiVO_4_ containing 5 wt% Ag showed a kinetic constant of 0.023 min^−1^, considerably higher than that of pure BiVO_4_ [70]. These examples support the trend observed in our Bi_1−x_Eu_x_VO_4_ series, where Eu^3+^ incorporation improves phase composition, enhances charge carrier separation, and ultimately increases the reaction rate during RhB degradation.

## 3. Discussion

### The Formation of ms- or tz-Crystalline Phase in Bi_1−x_Eu_x_VO_4_ (x = 0, 0.03, 0.06, 0.09, and 0.12) Samples

Recently, control over the crystalline phases, i.e., transformation from the *tz*-type to the *ms*-type, and the morphology of BiVO_4_ samples has been successfully obtained using microwave hydrothermal (MWHT) conditions without any template/surfactant, doping of metal ions, or pH change in the reaction solution [71]. Also, doping with RE ions such as Er^3+^, Yr^3+^, Gd^3+^, Sm^3+^, Tb^3+^, Nd^3+^, and Ce^3+^ resulted in phase transitions in BiVO_4_ and the significant improvement of photocatalytic properties [28,67,72,73,74,75,76,77,78,79]. A comparison of the literature on the formation of either *ms*- or *tz*-crystalline phases, as well as luminescent and photocatalytic properties as a function of the Eu^3+^/Bi^3+^ molar ratio in Bi_1−x_Eu_x_VO_4_ samples, is presented alongside our results in Table 4.

## 4. Materials and Methods

### 4.1. Materials

The following chemicals were used as received: bismuth nitrate pentahydrate, (Bi(NO_3_)_3_·5H_2_O (99%, Merck, Darmstadt, Germany), ammonium metavanadate, NH_4_VO_3_ (99%, Merck, Darmstadt, Germany), Europium (III) acetate hydrate, Eu(CH_3_COO)_3_·H_2_O (99.9%, Alfa Aesar, Haverhill, MA, USA), nitric acid, HNO_3_ (99%, Merck, Darmstadt, Germany), sodium hydroxide, NaOH (95%, Merck, Darmstadt, Germany), Rhodamine B, C_28_H_31_CIN_2_O_3_, and RhB (95%, Merck, Darmstadt, Germany).

### 4.2. Synthesis of Bi_1−x_Eu_x_VO_4_ (x = 0.03, 0.06, 0.09, and 0.12) Samples

In a typical microwave-assisted synthesis method, the aqueous solutions of ammonium metavanadate, NH_4_VO_3_ (6 mL, 0.05 M), bismuth(III) nitrate pentahydrate, and Bi(NO_3_)_3_·5H_2_O (5 mL, 0.05 M) were uniformly mixed in a 20 mL process vial equipped with a stirring bar. Additionally, the appropriate amount (0.03, 0.06, 0.09, and 0.12 mmol) of the respective europium (III) acetate hydrate, Eu(CH_3_COO)_3_·H_2_O, was added to the starting Bi(NO_3_)_3_ for each (of four) prepared solution individually. The reaction mixtures were heated in a closed vessel system of a microwave reactor at 170 °C for a duration of 10 min. The reaction mixtures were cooled down before being transferred to centrifugal tubes and then centrifuged for 20 min at 12,000 rpm to produce a light-yellow powder, which was washed three times using deionized water. Powder samples were finally obtained upon drying them in the air at 70 °C for three hours. The obtained powder samples in yellow color will be denoted through the text as Bi_0.97_Eu_0.03_VO_4_, Bi_0.94_Eu_0.06_VO_4_, Bi_0.91_Eu_0.09_VO_4_, and Bi_0.88_Eu_0.12_VO_4_, corresponding to 3 mmol, 6 mmol, 9 mmol, and 12 mmol of added Eu^3+^ ions in starting (Bi^3+^) solutions, respectively.

### 4.3. Synthesis of BiVO_4 4_samples

An undoped BiVO_4_ sample was prepared by applying identical reaction procedures of microwave synthesis as in the absence of Eu^3+^ precursors, Eu(CH_3_COO)_3_·H_2_O. Briefly, the reaction mixture containing aqueous solutions of NH_4_VO_3_ (6 mL, 0.05 M) and Bi(NO_3_)_3_·5H_2_O (5 mL, 0.05 M) was transferred into microwave reactor vial equipped with a stirring bar; it was heated in a closed vessel system at 170 °C for during 10 min, then cooled down to room temperature and centrifuged for 20 min to produce a light yellow powder that was washed three times using deionized water. A powder sample was finally obtained after drying at 70 °C for 3 h. The light-yellow powder of the BiVO_4_ sample has been denoted simply as BiVO_4_.

### 4.4. Characterization Instrumentation

A high-density microwave field chemical synthesis reactor, the Monowave 300 from Anton Paar GmbH (Graz, Austria), with a maximum magnetron output power of 850 W, was used for the microwave heating studies. With a working temperature of up to 300 °C, an integrated infrared temperature sensor was used to monitor the reaction temperature. PEEK snap caps and conventional silicone septa covered with polytetrafluoroethylene (PTFE) are used to seal the reusable 20 mL Pyrex vials (G30). Every experiment was conducted at a maximum pressure of 30 bar and a stirring rate of 600 rpm. The powder X-ray diffraction (XRD) analysis was performed using a BRUKER AXS GMBH A24A10 X-ray diffractometer (Bruker AXS GmbH, Karlsruhe, Germany). Patterns were collected at room temperature over a 2θ range of 15–70°, with a scan rate of 3°/min and a divergent slit of 0.5 mm, operating at 40 kV and 30 mA. Attenuated total reflectance Fourier transform infrared (ATR-FTIR) measurements were conducted in the 400–4000 cm^−1^ range, with a spectral resolution of 4 cm^−1^ at room temperature, using a Thermo Scientific Nicolet iS50 FT-IR spectrometer (Thermo Fisher Scientific, Waltham, MA, USA) equipped with a built-in all-reflective ATR diamond. In the 200–800 nm range, the Shimadzu 1800 UV–Vis spectrophotometer (Shimadzu Corporation, Kyoto, Japan) with a temperature controller was used to record the ultraviolet–visible (UV–Vis) absorption spectra. The Spectrophotometer Shimadzu UV–Visible UV-2600 (Shimadzu Corporation, Tokyo, Japan) with an integrated sphere (ISR-2600 Plus (for UV-2600)), with a 300–800 nm range and a 1 nm step, was used to quantify diffuse reflection. The morphology of the obtained samples was studied using a Tecnai F20 TEM/STEM microscope (FEI Company, now Thermo Fisher Scientific) at 200 kV electron acceleration voltage after the dry-transfer of the sample material on lacey carbon TEM grids. The UV–visible absorption spectrum was recorded using the spectrometer Quantaurus-QY, C11347 series, Hamamatsu (Hamamatsu Photonics, Hamamatsu City, Japan). Luminescence measurements were recorded using a Jobin Yvon (HORIBA Jobin Yvon, Edison, NJ, USA), Spex mod 1401, double grating monochromator with resolution in the visible region of 5 cm^−1^, in the photon counting measurement regime, exciting the samples with 375 nm laser diode oxxius mod. LBX-375-70-CSB-PP and 518 nm laser diode oxxius mod. LBX-515-150-CSB-PPA. Luminescence decay measurements were performed after excitation with the third harmonic of a pulsed Nd-YAG laser. The visible emission was collected using a double monochromator with a resolution of 5 cm^−1^, and the signal was analyzed using a photon-counting system. Decay curves were obtained by recording the signal with a multi-channel analyzer Stanford SR430 (Stanford Research Systems, Sunnyvale, CA, USA). A LabRAM HR Evolution confocal Raman microscope (Horiba France SAS, Palaiseau, France) was used to investigate the influence of Eu^3+^ doping on the crystal structure of BiVO_4_ samples. Raman spectra were recorded in backscattering configuration using a 532 nm continuous-wave (CW) laser operated at low power (1 mW). The laser was focused on the sample through a 100× microscope objective (NA = 0.9), and the scattered light was analyzed with a diffraction grating of 1800 lines/mm.

### 4.5. Photocatalytic Test

Photocatalytic activities of the as-prepared samples were determined through the decolorization of RhB under visible light irradiation. The experiments were conducted in a 100 mL glass reactor equipped with a 300 W lamp as the light source, positioned 30 cm from the reaction mixture. An Osram Vitalux lamp, OSRAM GmbH, Munich, Germany (300 W, white light: UVB radiated power from 280 to 315 nm 3.0 W; UVA radiated power 315–400 nm 13.6 W; the rest is visible light and IR) was used as the simulated sunlight source. Optical power was measured using an R-752 Universal Radiometer read-out with sensor model PH-30, DIGIRAD, and it was ∼30 mWcm^−2^.

In a standard experiment, 50 mL of RhB solution with a 5 ppm concentration was mixed with 40 mg of the appropriate Bi_1−x_Eu_x_VO_4_ (x = 0, 0.03, 0.06, 0.09, and 0.12) sample. Before illumination, the mixture was treated with an ultrasonic bath for 15 min and then left in the dark for one hour to reach adsorption–desorption equilibrium between RhB and the appropriate Bi_1−x_Eu_x_VO_4_ sample. At given time intervals, the collected mixture samples (1 mL) were centrifuged at 12,000 rpm for 20 min to remove the catalyst. The sample concentration was determined by recording the absorbance at 554 nm using a UV-1800 spectrophotometer (Shimadzu). The degradation efficiency (D) was calculated using Equation (4):

(4) $$D\left(\%\right)=\left(\frac{C_{0}-C_{t}}{C_{0}}\right)\times 100$$

where C_0_ is the initial concentration of the dye solution, and C_t_ is the concentration of the dye solution after a certain time of illumination (t). 

## 5. Conclusions

In this manuscript, a highly effective, controlled, and eco-friendly microwave-assisted approach was developed for preparing nanostructured Bi_1−x_Eu_x_VO_4_ (x = 0, 0.03, 0.06, 0.09, and 0.12) with enhanced photoluminescent and photocatalytic properties. The effects of the concentration of Eu^3+^ ions integrated into the BiVO_4_ matrix on the formation of the *ms*- or *tz*-type BiVO_4_ structure, photoluminescent intensity, decay dynamics of europium emission, and photocatalytic effectiveness in RhB degradation were thoroughly examined. The main aim was to obtain the optimized *tz*-BiVO_4_ nanostructure, which had been presented in our recently published papers as a structure exhibiting better photocatalytic performance than *ms*-BiVO_4_ with respect to pollutant removal.

The FTIR analysis of Bi_1−x_Eu_x_VO_4_ (x = 0.03, 0.06, 0.09, and 0.12) samples showed the characteristic peaks as undoped BiVO_4_ in the range of around 500–820 cm^−1^, while two new peaks appeared at 471 cm^−1^ and 720 cm^−1^, assigned to Eu-O stretching vibrations. The band gap, Eg, estimated from the absorption edge wavelength of the inter-band transition, shifted to higher energy with increasing Eu^3+^ contents and ranged from approximately 2.55 to 2.80 eV. The PL spectra of samples that contained Eu^3+^ ions displayed dominant emissions corresponding to the ^5^D_0_ → ^7^F_2_ transition, whereas BiVO_4_ does not show any emissions. Also, the Eu^3+^-doped BiVO_4_ exhibited a much higher photocatalytic activity in the degradation of RhB than undoped BiVO_4_.

In our future works, research will be focused on the photocatalytic degradation of other organic pollutants when using the Eu^3+^-doped BiVO_4_ photocatalyst and different rare earth/dopant ions in a wide range of their concentrations.

## Figures and Tables

**Table 1:** Recorded fluorescence decays corresponding to the ^5^D_0_ ⟶ ^7^F_2_ transition for Bi_1−x_Eu_x_VO_4_ (x = 0.03, 0.06, 0.09, 0.12) samples under 355 nm pulsed excitation.

Sample	*τ* Lifetime [ns]	1/e Lifetime [ns]	Average Lifetime [µs]
Bi_0.97_Eu_0.03_VO_4_	80	2868	60
Bi_0.94_Eu_0.06_VO_4_	<10	2675	100
Bi_0.91_Eu_0.09_VO_4_	<10	1280	30
Bi_0.88_Eu_0.12_VO_4_	<10	1638	36

**Table 2:** Raman peak positions and vibrational mode assignments in Bi_1−x_Eu_x_VO_4_ samples, showing contributions from monoclinic (*ms*) and tetragonal (*tz*) phases.

Wavelength [cm^−1^]	Description	Attribution
(a) 179	External lattice mode	*ms*-BiVO_4_ distortions
(b) 197	External lattice mode	*ms*-BiVO_4_ distortions
(c) 211	External lattice mode	*ms/tz*-BiVO_4_
(d) 247	Bi–O stretching mode	*tz*-BiVO_4_
(e) 327	Symmetric bending mode of VO_4_^3^	*ms/tz*-BiVO_4_
(f) 367	Asymmetric bending mode of VO_4_^3^	*ms/tz*-BiVO_4_
(g) 708	Asymmetric stretching mode of V-O bond	*ms*-BiVO_4_
(h) 778	Antisymmetric stretching mode V-O bond	*tz*-BiVO_4_
(i) 829	Symmetric stretching mode of V-O	*ms*-BiVO_4_
(j) 854	Symmetric stretching mode V-O bond	*tz*-BiVO_4_

**Table 3:** Apparent pseudo-first-order rate constants $k_{\mathrm{app}}$ and correlation coefficients R^2^ for RhB photodegradation over Bi_1−x_Eu_x_VO_4_ photocatalysts under visible light irradiation.

Sample	$k_{\mathrm{app}}$ (min^−1^)	R^2^	$k_{\mathrm{app}}/k_{{\mathrm{BiVO}}_{4}}$
BiVO_4_	1.67 × 10^−2^	0.9678	1.00
Bi_0.97_Eu_0.03_VO_4_	2.26 × 10^−2^	0.9684	1.35
Bi_0.94_Eu_0.06_VO_4_	1.84 × 10^−2^	0.9511	1.10
Bi_0.91_Eu_0.09_VO_4_	2.21 × 10^−2^	0.9863	1.33
Bi_0.88_Eu_0.12_VO_4_	3.46 × 10^−2^	0.9508	2.07

**Table 4:** Literature comparison of the formation of *ms*- or *tz*-crystalline phase and luminescent and photocatalytic properties of Bi_1−x_Eu_x_VO_4_ samples with results of this work.

Sample/ Method of Synthesis	xEu^3+^ x = conc.(Eu^3+^) (mmol)	Crystalline Phase	Luminescent and Photocatalytic Properties	Ref.
Eu^3+^-uniformly-doped BiVO_4_ NPs *	x = 0.0–0.5	*ms*	^5^D_0_ → ^7^F_0,1,2,3,4_ PL quenched; cutoff edge 530 nm and ~518 nm.	
	x = 0.6–0.9	Mixture *ms*-*tz*	Blue shift appears of the absorption edges.	[65]
	x = 0.9–1.0	*tz*	^5^D_0_ → ^7^F_0,1,2,3,4_ PL observed.Blue shift appears of the absorption edges.	
Eu^3+^-surface- localized BiVO_4_ NPs	x = 0~0.6	*ms*	Enhanced Eu^3+^-PL and improved photocatalysis.	[65]
Bi_x_Eu_1−x_VO_4_	0 < x < 0.60	*tz*	DRS: The broad bands attributed to charge transfer processes. The sharp peaks are ascribed to intra-configurational 4f–4f transitions of the Eu^3+^ ion in Bi_x_Eu_1−x_VO_4_.	[80]
	0.94 < x < 1	*ms*		
EuVO_4_–BiVO_4_	0.35 < x < 0.70	*tz*	-	[81]
	0.75 < x < 0.90	Mixture *ms-tz*	-	
Eu_1−x_Bi_x_VO_4_/P	x = 0.05	*tz*	PL: The energy transfer and modification of the lifetime of the electron/hole pair formation of the Eu_1−x_Bi_x_VO_4_; higher photocatalytic degradation efficiency of MB compared to the undoped material.	[59]
Eu_1−x_Bi_x_VO_4_/MWHT	x = 0.05	*ms*		
Eu_1−x_Bi_x_VO_4_/P-HT	0 < x < 1	*tz*	Strong red emission under both near-UV and Vis excitation.	[82]
Bi_x_Eu_1−x_VO_4_/SG	x = 0, 0.01, 0.03, 0.05, 0.07 and 0.10	*ms*	DRS: Reduction in Eg from 2.43 to 2.38 eV with Eu^3+^ doping, indicating the formation of new low-energy-level transitions within the band gap; Eu^3+^ doping significantly improved photocatalytic efficiency.	[83]
Bi_x_Eu_1−x_VO_4_/NPss/SC	x = 0.5, 1.0 and 1.5	*ms*	PL: An intense red emission at 615 nm under excitation wavelength with 266 and 355 nm.	[84]
Bi_x_Eu_1−x_VO_4_/MW	x = 0	*ms*	PL: The Bi_1−x_EuxVO_4_ samples display dominant emission corresponding to ^5^D_0_ → ^7^F_2_ transition; the BiVO_4_ does not show any emission. Photocalysis: The Bi_1−x_EuxVO_4_ samples exhibit much higher photocatalytic activity in the degradation of RhB than undoped BiVO_4_.	**This work**
	x = 0.3, 0.06, 0.09	Mixture *ms-tz*		
	x = 0.12	Dominant *tz*		

* *ms*—monoclinic scheelite structure; *tz*—tetragonal zircon structure; NPs—nanoparticles; PL—photoluminescence; DRS—diffuse reflectance spectroscopy; P—precipitation; MWHT—microwave-assisted hydrothermal; P-HT—precipitation–hydrothermal; MB—methylene blue; SG—sol–gel; UV—ultraviolet; Vis—visible; Eg—energy gap; SC—solution combustion; MW—microwave.

## Data Availability

The original contributions presented in this study are included in the article. Further inquiries can be directed to the corresponding authors.
